# De‐Epithelialization Using Scalpel Versus Monopolar Electrocautery Cutting in Reduction Mammoplasty: A Prospective Comparative Study

**DOI:** 10.1002/wjs.70574

**Published:** 2026-09-15

**Authors:** Khaled E. Barakat, Mohamed F. Asal, Ahmed A. Abdelkader, Doaa K. Abuhasan, Anas Bakdalieh, Annaliisa Cordova, Adil Nusair, Ahmed Adham R. Elsayed, Marc D. Basson

**Affiliations:** ^1^ Surgical Oncology Unit Surgery Department Faculty of Medicine Alexandria University Alexandria Egypt; ^2^ New Borg Al Arab Hospital Directorate of Health Affairs in Alexandria Ministry of Health and Population Alexandria Egypt; ^3^ Medical Education Department Faculty of Medicine Alexandria University Alexandria Egypt; ^4^ College of Medicine Northeast Ohio Medical University Rootstown Ohio USA; ^5^ Department of Surgery Northeast Ohio Medical University Rootstown Ohio USA; ^6^ Department of Biomedical Sciences Northeast Ohio Medical University Rootstown Ohio USA

**Keywords:** de‐epithelialization, monopolar electrocautery, reduction mammoplasty, scalpel

## Abstract

**Background:**

Reduction mammoplasty for symptomatic macromastia provides meaningful relief from musculoskeletal pain, postural strain, and functional limitations. A critical step in the procedure is pedicle de‐epithelialization, traditionally performed using a scalpel. Although effective, scalpel de‐epithelialization can be time‐intensive, prompting evaluation of alternative techniques such as monopolar electrocautery. Other sharp techniques include curved‐scissor de‐epithelialization, en bloc removal, and pedicle deskinning. Monopolar techniques utilize either a conventional blade electrode or a Colorado needle tip. This study compares surgical outcomes between scalpel and monopolar electrocautery de‐epithelialization.

**Methods:**

A prospective, intra‐patient comparative study was conducted at the Department of Surgery, Alexandria Faculty of Medicine, between October 2023 and April 2025. Patients with symptomatic macromastia undergoing bilateral reduction mammoplasty received scalpel de‐epithelialization on the right breast and monopolar electrocautery on the left. Follow‐up assessments occurred at 1 week, 2 weeks, 1 month, and monthly thereafter for up to 12 months. The primary outcome was de‐epithelialization time. Secondary outcomes were intraoperative blood loss and pain both postoperatively and at 6 months. Statistical analyses included paired *t*‐tests, Wilcoxon signed‐rank tests, and McNemar's tests.

**Results:**

Sixty patients undergoing bilateral reduction mammoplasty were included. Monopolar electrocautery demonstrated a significantly shorter mean de‐epithelialization time compared to the scalpel (5.07 ± 0.88 minutes vs. 10.87 ± 1.93 minutes; *p* < 0.001). Intraoperative blood loss was also significantly lower with monopolar electrocautery (139.1 ± 20.64 mL vs. 176.7 ± 16.06 mL; *p* < 0.001). At 6 months, BREAST‐Q breast pain scores were modestly lower with the monopolar technique (*p* = 0.046). Rates of postoperative complications and patient satisfaction were comparable between techniques.

**Conclusions:**

Monopolar electrocautery significantly reduced de‐epithelialization time, the primary outcome of this study. It was also associated with lower intraoperative blood loss. Although modestly lower breast pain scores were observed at 6 months, the difference was small, and its clinical importance remains uncertain. The study was not powered to evaluate postoperative complication rates.

## Graphical abstract

Monopolar electrocautery offers an efficient alternative to scalpel de‐epithelialization in reduction mammoplasty, significantly reducing operative time and blood loss while maintaining comparable observed postoperative and patient‐reported outcomes.

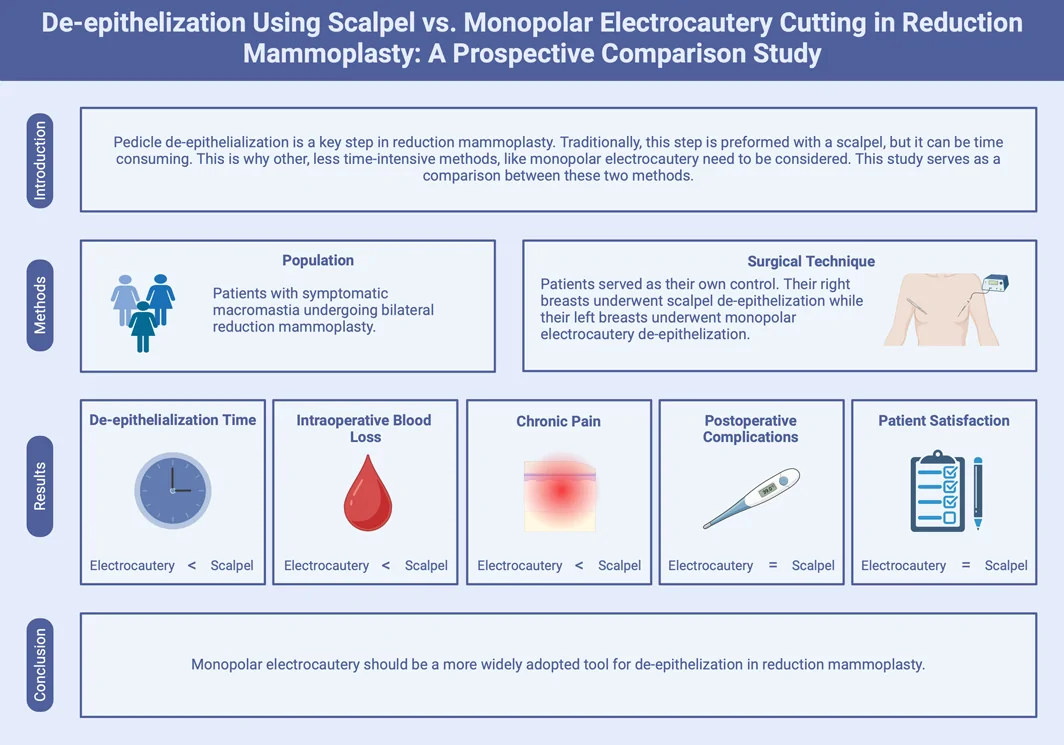

## 1 Introduction

Musculoskeletal pain, postural strain, skin irritation, and functional impairment from symptomatic macromastia may substantially reduce quality of life [1, 2]. Macromastia may also cause psychosocial distress, including low self‐esteem, embarrassment arising from breast‐focused comments, and fear of sexual harassment [1]. 100,000 women undergo reduction mammoplasty annually [3]. Reduction mammoplasty involves resection of excess breast tissue and skin with nipple–areolar complex (NAC) preservation and repositioning, aiming to relieve symptoms while restoring proportion and breast shape [4, 5]. Reduction mammoplasty may be preferred over conservative management of macromastia because surgery consistently alleviates physical symptoms, improves functional capacity, and durably improves health‐related quality of life [6].

Many reduction mammoplasty approaches require pedicle de‐epithelialization. This removes the epidermis while preserving the underlying dermis and its subdermal vascular plexus, thereby maintaining perfusion and structural integrity for the pedicle and NAC [7]. De‐epithelialization is traditionally performed by careful shallow scalpel dissection immediately beneath the basal layer, protecting the dermal blood supply [8]. Curved‐scissor de‐epithelialization is an alternative particularly useful for long pedicles in which the marked skin is divided into narrow sections [9]. This is contrary to en bloc removal, in which the epidermis is lifted as one continuous sheet [10]. Pedicle deskinning is another technique mainly used in inferior‐pedicle reductions. This removes both the epidermis and dermis when dermal preservation unnecessary [11]. Monopolar de‐epithelialization using a Colorado needle tip has also been described for de‐epithelialization of keyhole skin patterns [12]. Although effective, scalpel de‐epithelialization takes time and slows the procedure. The time burden and the need for meticulous tissue handling have motivated interest in alternative approaches, such as deskinning, which was reported to take only 6 minutes [11]. Other techniques, including monopolar electrocautery, may accelerate de‐epithelialization while maintaining acceptable surgical safety profiles [13, 14].

We hypothesized that replacing the scalpel with monopolar electrocautery would reduce surgical time and blood loss without adversely affecting patient outcomes. This prospective comparative study used a within‐subject design to compare scalpel and monopolar electrocautery de‐epithelialization in bilateral reduction mammoplasty, with outcomes recorded separately for each breast.

## 2 Material and Methods

### 2.1 Study Design and Setting

This prospective, intra‐patient comparative study was conducted at the Department of Surgery, Alexandria University Faculty of Medicine, from October 2023 to April 2025. Under this design, scalpel de‐epithelialization was performed on one breast and cutting monopolar electrocautery on the contralateral breast during the same procedure. Because both breasts were treated under identical perioperative conditions, paired statistical analyses were used to compare breast‐specific outcomes while controlling for patient‐level differences. The study was approved by the institutional review boards of Alexandria University Faculty of Medicine and Northeast Ohio Medical University. Written informed consent was obtained from all participants after they received detailed information about both procedures and associated risks and benefits. The study was conducted in accordance with the principles of the Declaration of Helsinki and Good Clinical Practice.

### 2.2 Patient Selection

We assessed consecutive patients with symptomatic macromastia undergoing bilateral reduction mammoplasty for inclusion. Eligibility criteria included: age 18–60 years, BMI ≤ 35 kg/m^2^, sternal notch‐nipple distance < 35 cm, symmetrical breast hypertrophy requiring similar volume reductions bilaterally, and no previous breast surgery or radiotherapy. Patients with heavy smoking, uncontrolled diabetes mellitus, history of chest wall irradiation or previous breast surgery, known coagulopathies or autoimmune disease, and notable breast asymmetry forbidding the two‐sided proportionate reduction were excluded. All consecutive patients who fulfilled these criteria were consented to and enrolled; no eligible and consenting patients were excluded.

### 2.3 Sample Size Calculation

The primary outcome of interest was time to de‐epithelialization. The sample size was calculated accordingly. Preliminary institutional data from Alexandria University showed a reduction in operative time from 14 minutes with scalpel to 3.5 minutes with monopolar electrocautery, a 75% reduction. These data were used only for the sample size calculation, and the corresponding patients were not included in the current study. A sample size of 54 patients provided 80% power, with a two‐tailed alpha of 0.05 and a standard deviation of approximately 2 minutes, to detect this difference. Allowing for a 10% dropout rate, 60 patients were recruited. Sample size was calculated using G*Power software (Version 3.1.9.7, Düsseldorf, Germany).

### 2.4 Surgical Technique

All procedures were performed under general anesthesia by either M.F.A or A.A.A to minimize inter‐operator variability. Before surgery, the areas to be de‐epithelialized were marked using conventional reduction mammoplasty markings. Patients were assigned to scalpel de‐epithelialization on the right breast and monopolar electrocautery on the left. The right breast was consistently operated on first in all cases. The assignment of technique to side was fixed consistently across all patients rather than randomized. This decision was made deliberately for several operational reasons. This protocol standardized the procedural sequence across all cases, facilitated data collection, and ensured seamless correlation between intraoperative data recorded in the operating room and outcome data collected during postoperative clinic follow‐up visits. The fixed right/left convention allowed unambiguous linkage of each breast's data across all time points. Patients were blinded to the technique assignment, but blinding of the operating surgeons was not feasible given the inherently distinct visual and tactile characteristics of the two methods.

Scalpel de‐epithelialization was performed using a No. 11 or 15 blade with sharp dissection immediately beneath the basal layer while preserving the subdermal vascular plexus. For monopolar electrocautery de‐epithelialization, cut diathermy was used with standardized settings while preserving the dermal blood supply.

Both superomedial pedicle (primary technique in 95% of cases) and inferior pedicle (5% of cases) techniques were performed according to preoperative planning, depending on patient anatomy. The rest of the reduction mammoplasty procedure (i.e., pedicle isolation, excess tissue resection, and wound closure) was performed uniformly on both sides. The operating surgeon completed the main operative steps on the right breast before proceeding to the left, while the assistant completed hemostasis, drain placement as indicated, and wound closure on the first side. Because of this overlapping workflow, total bilateral skin‐to‐skin operative time was less than the sum of the individual breast operative intervals.

## 3 Data Collection and Outcome Measures

The primary outcome was time to de‐epithelialization (minutes) for each breast, measured from the first incision of the de‐epithelialization step to its completion. Total single‐side operative time was also assessed as a secondary outcome. Other secondary outcomes included intraoperative blood loss, postoperative pain, postoperative complications, and BREAST‐Q outcomes. Timing was recorded using a dedicated stopwatch by a single designated scrub nurse present throughout all procedures. The start and end points were verbally announced by the operating surgeon to standardize the recording interval. Total single‐side operative time was defined as the time from skin incision to completion of wound closure for each breast separately and was recorded by the same designated scrub nurse. Because operative work on the two breasts overlapped, these breast‐specific intervals were not additive. This metric was used to assess technique‐specific efficiency for each breast and does not represent total bilateral skin‐to‐skin operative time or anesthesia time. Intraoperative blood loss was quantified using gravimetric and volumetric methods as previously described [15]. Surgical sponges were weighed before and after use using a calibrated digital scale, with blood volume calculated from the weight difference assuming a blood density of 1.06 g/mL. Suction canister contents were measured volumetrically after subtracting irrigation fluid volumes. Postoperative pain was assessed separately for each breast using a 10‐cm Visual Analogue Scale (VAS), ranging from 0 (no pain) to 10 (worst imaginable pain). VAS assessments were performed at rest, at a standardized time (09:00–10:00 AM) on postoperative days 1 and 3, by a study nurse who was not part of the operating team. Patients were instructed to rate pain localized to each breast independently. Analgesic administration followed a standardized postoperative protocol applied uniformly to all patients, and no additional analgesics were administered within 2 hours preceding each VAS assessment. We also recorded complications (infection, hematoma, seroma, partial or complete nipple‐areolar complex necrosis, wound dehiscence, flap necrosis), and aesthetic and satisfaction outcomes as assessed with the BREAST‐Q Reduction Module. All operative measurements were performed by the same specialized research nurse across all cases to minimize interobserver variability. The BREAST‐Q was administered twice to each patient, once for each breast separately, with explicit instructions to answer each item with either the right or the left breast in mind, as appropriate. This approach was confirmed as acceptable by the BREAST‐Q support team (Q‐Portfolio Team), who confirmed that the instrument may be used twice per patient for research purposes, once per breast.

### 3.1 Postoperative Assessment and Follow‐Up

In the immediate postoperative period, we monitored for infection, hematoma, bleeding, and complications related directly to the wound, as well as the viability of the nipple‐areolar complex. Wound healing issues were defined as wound dehiscence, delayed healing (epithelialization not complete by 4 weeks), superficial skin necrosis outside the NAC, or incision‐related breakdown requiring treatment beyond routine wound care. Partial NAC necrosis was evaluated separately under NAC viability because it reflects a perfusion‐related complication of the pedicle rather than a primary wound healing problem. Hematoma was defined as any blood collection requiring evacuation. Infection was defined as wound erythema with purulent discharge or cellulitis requiring antibiotic therapy or surgical drainage.

Follow‐up evaluations were performed for 1 week and 2 weeks postoperatively, at 1 month, and subsequently monthly until 12 months.

### 3.2 Statistical Analysis

All continuous variables were analyzed with a paired *t*‐test in case of normal distribution and Wilcoxon signed‐rank tests in case of non‐normal distribution. For categorical variables, comparisons were made using McNemar's test. A *p* ≤ 0.05 was the cut‐off for statistical significance. Statistical analysis of the data was performed using IBM SPSS Statistics version 26.

## 4 Results

Sixty patients undergoing bilateral reduction mammoplasty were enrolled in this prospective comparative study. The study cohort comprised 57 women with superomedial pedicle reduction (95%) and 3 women with inferior pedicle technique (5%). Specimen weights were comparable between the two breasts. Mean sternal notch‐to‐nipple distance was 32.15 ± 1.89 cm (Table 1).

**TABLE 1:** Distribution of the studied cases according to patient characteristics (*n* = 60).

	No. (%)
Sternal notch to nipple (cm)	
Min.—Max.	28.0–35.0
Mean ± SD.	32.15 ± 1.89
Median (IQR)	32.0 (31.0–34.0)
Age (years)	
Min.—Max.	25.0–59.0
Mean ± SD.	46.13 ± 7.38
Median (IQR)	46.0 (42.0–51.0)
BMI	
Min.—Max.	22.80–34.80
Mean ± SD.	28.86 ± 3.42
Median (IQR)	28.45 (26.55–31.45)
Specimen weight for right breast side (gm)	
Min.—Max.	790–1100
Mean ± SD.	902.84 ± 57.85
Median (IQR)	910 (867.5–940)
Specimen weight for left breast side (gm)	
Min.—Max.	810–1170
Mean ± SD.	951.16 ± 65.87
Median (IQR)	960 (917.5–990)
Total operative time (min)	
Min.—Max.	127–183
Mean ± SD.	150.58 ± 12.56
Median (IQR)	150 (140.75–159)
Comorbidities	
No	40 (66.7%)
DM	10 (16.7%)
HTN	4 (6.7%)
HTN + DM	6 (10.0%)
Smoking	
No	58 (96.7%)
Yes	2 (3.3%)
Side of the scalpel device (right)	60 (100.0%)
Side of the monopolar device (left)	60 (100.0%)
Type of pedicle	
Superomedial	57 (95.0%)
Inferior	3 (5.0%)

Abbreviations: IQR, Interquartile range; SD, Standard deviation.

De‐epithelialization time was significantly shorter with monopolar electrocautery, corresponding to a 53.4% reduction compared with the scalpel technique (*p* < 0.001). Total single‐sided operative time was also significantly shorter with monopolar electrocautery, with a mean reduction of approximately 9 minutes (*p* < 0.001) (Table 2).

**TABLE 2:** Comparison between the two methods according to surgical outcomes.

	Scalpel (*n* = 60)	Monopolar (*n* = 60)	Test of Sig.	*p*
De‐epithelialization time (min)				
Min.—Max.	8.0–18.0	4.0–8.0	Z = 6.766^*^	< 0.001^*^
Mean ± SD.	10.87 ± 1.93	5.07 ± 0.88		
Median (IQR)	10.50 (9.50–12.0)	5.0 (4.0–6.0)		
Single side operative time (min)				
Min.—Max.	88.0–130.0	75.0–124.0	Z = 6.809^*^	< 0.001^*^
Mean ± SD.	98.67 ± 8.25	89.57 ± 9.37		
Median (IQR)	97.50 (93.0–102.0)	89.0 (83.0–92.50)		
Blood loss (mL)				
Min.—Max.	143.0–211.0	37.0–174.0	*t* = 11.825^*^	< 0.001^*^
Mean ± SD.	176.7 ± 16.06	139.1 ± 20.64		
Median (IQR)	178.0 (165.0–188.5)	138.5 (130.0–149.0)		
Pain scores day 1				
Min.—Max.	3.0–5.0	3.0–5.0	Z = 0.474	0.635
Mean ± SD.	3.65 ± 0.66	3.60 ± 0.62		
Median (IQR)	4.0 (3.0–4.0)	4.0 (3.0–4.0)		
Pain scores day 3				
Min.—Max.	2.0–3.0	1.0–4.0	Z = 0.243	0.808
Mean ± SD.	2.15 ± 0.36	2.13 ± 0.43		
Median (IQR)	2.0 (2.0–2.0)	2.0 (2.0–2.0)		

Abbreviations: IQR, Interquartile range; p, *p*‐value for comparing between the two periods; SD, Standard deviation; *t*, Paired *t*‐test; Z, Wilcoxon signed‐rank test.

^*^ Statistically significant at *p* ≤ 0.05.

Intraoperative blood loss was significantly lower with monopolar electrocautery, with a mean reduction of 37.6 mL compared to scalpel de‐epithelialization (*p* < 0.001). Pain scores measured on the Visual Analog Scale were comparable between groups on postoperative days 1 and 3 (Table 2).

Postoperative complications were rare and numerically comparable between techniques. Partial nipple‐areolar complex necrosis occurred in 2 patients (3.3%) in each group. Hematoma developed in 2 patients (3.3%) with the scalpel technique and none with the monopolar technique. Infection occurred in 1 patient (1.7%) with the monopolar and 2 patients (3.3%) with the scalpel. No patients experienced flap necrosis or wound healing issues in either group (Table 3). No statistically significant differences were detected for any complication; however, as detailed in the limitations, the study was not adequately powered to draw conclusions regarding complication equivalence, and these findings should be interpreted accordingly.

**TABLE 3:** Distribution of the studied cases according to postoperative complications.

	Scalpel (*n* = 60)	Monopolar (*n* = 60)	^McN^ *p*
Post‐operative complications			
NAC viability (partial necrosis)	2 (3.3%)	2 (3.3%)	1.000
Flap necrosis	0 (0.0%)	0 (0.0%)	—
Hematoma	2 (3.3%)	0 (0.0%)	0.500
Infection	2 (3.3%)	1 (1.7%)	1.000
Wound healing issues	0 (0.0%)	0 (0.0%)	—

Abbreviations: McN, McNemar test; p, *p*‐value for comparing between the two periods.

Regarding 6‐month Breast‐Q outcomes, breast pain was slightly lower with the monopolar technique (*p* = 0.046). Patient satisfaction with surgical outcomes was high and comparable between groups. Subjective satisfaction with nipple positioning, alignment, shape, appearance, and sensation showed no significant differences between techniques (Table 4).

**TABLE 4:** Comparison between the two methods according to the Breast Q reduction module.

	Scalpel (*n* = 60)	Monopolar (*n* = 60)	Z	*p*
Pain breast				
Min.—Max.	2.0–3.0	2.0–3.0	2.000	0.046^*^
Mean ± SD.	2.87 ± 0.34	2.80 ± 0.40		
Median (IQR)	3.0 (3.0–3.0)	3.0 (3.0–3.0)		
Satisfaction with breasts				
Min.—Max.	75.0–100.0	75.0–100.0	0.962	0.336
Mean ± SD.	88.77 ± 7.30	89.17 ± 7.38		
Median (IQR)	86.0 (82.0–92.0)	89.0 (82.0–92.0)		
Satisfaction with outcome				
Min.—Max.	76.0–100.0	76.0–100.0	1.131	0.258
Mean ± SD.	89.47 ± 8.82	90.10 ± 8.83		
Median (IQR)	86.0 (86.0–100.0)	86.0 (86.0–100.0)		
Satisfaction with nipples				
High low				
Min.—Max.	1.0–4.0	1.0–4.0	0.447	0.655
Mean ± SD.	3.47 ± 0.68	3.48 ± 0.68		
Median (IQR)	4.0 (3.0–4.0)	4.0 (3.0–4.0)		
Lined up				
Min.—Max.	1.0–4.0	1.0–4.0	0.711	0.477
Mean ± SD.	3.65 ± 0.66	3.70 ± 0.65		
Median (IQR)	4.0 (3.0–4.0)	4.0 (4.0–4.0)		
Shape				
Min.—Max.	1.0–4.0	1.0–4.0	1.310	0.190
Mean ± SD.	3.50 ± 0.75	3.40 ± 0.74		
Median (IQR)	4.0 (3.0–4.0)	4.0 (3.0–4.0)		
Look				
Min.—Max.	1.0–4.0	1.0–4.0	0.702	0.483
Mean ± SD.	3.52 ± 0.68	3.55 ± 0.67		
Median (IQR)	4.0 (3.0–4.0)	4.0 (3.0–4.0)		
Sensation				
Min.—Max.	1.0–4.0	1.0–4.0	0.557	0.577
Mean ± SD.	3.40 ± 0.72	3.43 ± 0.72		
Median (IQR)	3.50 (3.0–4.0)	4.0 (3.0–4.0)		

Abbreviations: IQR, Interquartile range; p, *p*‐value for comparing between the two periods; SD, Standard deviation; Z, Wilcoxon signed‐rank test.

^*^ Statistically significant at *p* ≤ 0.05.

## 5 Discussion

While scalpel de‐epithelialization is effective with high patient satisfaction scores overall, it is frequently time‐intensive. Therefore, this study compared the common approach of scalpel de‐epithelialization against monopolar electrocautery cutting. Monopolar electrocautery reduced de‐epithelialization time, one‐sided operative time, operative blood loss, and pain at 6 months compared to scalpel de‐epithelialization.

Monopolar electrocautery cutting significantly shortened de‐epithelialization time and single‐sided operative time. Electrocautery is routinely used for hemostasis during reduction mammoplasty, but extending its use to de‐epithelialization may improve operative efficiency by reducing instrument exchanges and interruptions for bleeding control [16]. Pedicle de‐epithelialization is one of the more time‐consuming components of reduction mammoplasty, so shortening this step would be expected to translate into shorter overall operative duration [7]. The observed scalpel de‐epithelialization times in our study occurred in a cohort characterized by long sternal notch‐to‐nipple distances, which enlarge the pedicle surface area and predict longer de‐epithelialization times independent of instrument choice. The observed reduction in total single‐sided operative time exceeded the reduction in de‐epithelialization time alone. This additional time savings may be related to the coagulating action of monopolar electrocautery, reducing hemostatic pauses and the need for repeated instrument exchanges between scalpel and electrocautery [16].

The relatively long single‐side operative times in this study reflect the combination of symptomatic macromastia, long sternal notch‐to‐nipple distances, and large specimen weights, which together required more extensive pedicle preparation, parenchymal resection, and contouring than smaller reductions. Nevertheless, the mean total bilateral skin‐to‐skin operative time was approximately 151 minutes because several operative steps overlapped between the two sides. The overall bilateral operative time was consistent with operative times reported in the literature for reduction mammoplasty [17]. Longer reduction mammoplasty times have been associated with higher complication rates, including pneumonia and wound infection, while shorter cases reduce anesthesia exposure and operating‐room resource utilization [18].

Monopolar electrocautery de‐epithelialization had less overall blood loss compared to the scalpel technique. When dissection and coagulation occur in conjunction [19], this can reduce bleeding from the subdermal vascular plexus and improve operative field visibility, with fewer pauses required for hemostasis [20, 21]. Increased blood loss can also predispose patients to postoperative hematomas and distortion of the postsurgical result [22].

Although we observed no difference in early postoperative pain, monopolar electrocautery was associated with less pain on the 6‐month BREAST‐Q compared to scalpel de‐epithelialization. Monopolar electrocautery induces cell vapourization, leading to immediate necrosis of the localized sensory nerves [23]. This disrupts the injured nerves' impulses while maintaining the integrity of nearby structures [23, 24]. This process may be similar to that which causes third‐degree burns to experience anesthesia [25]. The main indication for reduction mammoplasty is relief from chronic pain involving the patient's back, neck, and shoulders [26]. Chronic pain can also have psychological effects, with people with chronic back pain being 6.2 times more likely to be depressed than those who are pain‐free [27, 28]. However, the absolute difference in mean breast pain score was small and is unlikely to be clinically important. These findings suggest that monopolar electrocautery was not associated with worse chronic breast pain.

One limitation of this study was its small sample size. However,the study was powered to detect a clinically meaningful difference in the primary outcome of de‐epithelialization time. The study was still underpowered to detect differences in postoperative complications, although these events were relatively rare. The lack of statistically significant differences in complications should therefore be interpreted with caution. Another limitation was that follow‐up was limited to 12 months, which prevented longer‐term outcomes evaluation. This was also a single‐center study, which limits the generalizability of the findings. While patients were blinded to technique assignment, the operating surgeons could not be blinded given the differences between the two methods. This introduces the potential for performance and observer bias. Additionally, histological assessment was not performed, so subclinical thermal effects on dermal microarchitecture, including hypertrophic scarring or fibrosis, cannot be excluded. Finally, the fixed, non‐randomized assignment of technique to each side introduces potential laterality bias, while consistently operating on the right breast first may introduce sequence bias.

## 6 Conclusion

Monopolar electrocautery de‐epithelialization demonstrated meaningful improvements in operative efficiency by significantly reducing de‐epithelialization time, the primary outcome of this study. It was also associated with lower intraoperative blood loss. Although a small improvement in 6‐month breast pain was observed, the clinical importance of this finding remains uncertain. This study was not powered to determine differences in postoperative complications, and larger studies are warranted to evaluate safety outcomes.

## Author Contributions

**Khaled E. Barakat:** conceptualization, methodology, data curation, investigation, formal analysis, writing–original draft, writing–review and editing. **Mohamed F. Asal:** conceptualization, methodology, data curation, investigation, formal analysis, writing–original draft, writing–review and editing. **Ahmed A. Abdelkader:** conceptualization, investigation, writing–original draft, writing–review and editing, methodology. **Doaa K. Abuhasan:** methodology, writing–original draft, writing–review and editing, formal analysis. **Anas Bakdalieh:** visualization, writing–original draft, writing–review and editing. **Annaliisa Cordova:** visualization, writing–original draft, writing–review and editing. **Adil Nusair:** visualization, writing–original draft, writing–review and editing. **Ahmed Adham R. Elsayed:** visualization, writing–original draft, writing–review and editing. **Marc D. Basson:** visualization, writing–original draft, writing–review and editing, supervision.

## Funding

The authors have nothing to report.

## Ethics Statement

The study was approved by the institutional review boards of Alexandria University Faculty of Medicine and Northeast Ohio Medical University.

## Conflicts of Interest

The authors declare no conflicts of interest.

## Data Availability

The data that support the findings of this study are available from the corresponding author upon reasonable request.
